# A Third Way of Energy Conservation in Acetogenic Bacteria

**DOI:** 10.1128/spectrum.01385-22

**Published:** 2022-06-14

**Authors:** Florian Kremp, Jennifer Roth, Volker Müller

**Affiliations:** a Department of Molecular Microbiology and Bioenergetics, Institute of Molecular Biosciences, Johann Wolfgang Goethe University, Frankfurt, Germany; University of Southern Denmark

**Keywords:** methylene-THF reductase, acetogenesis, bioenergetics, energy conservation, periplasmic hydrogenase

## Abstract

Acetogenic bacteria are a group of strictly anaerobic bacteria that make a living from acetate formation from two molecules of CO_2_ via the Wood-Ljungdahl pathway (WLP). The free energy change of this reaction is very small and allows the synthesis of only a fraction of an ATP. How this pathway is coupled to energy conservation has been an enigma since its discovery ~90 years ago. Here, we describe an electron transport chain in the cytochrome- and quinone-containing acetogen Sporomusa ovata that leads from molecular hydrogen as an electron donor to an intermediate of the WLP, methylenetetrahydrofolate (methylene-tetrahydrofolate [THF]), as an electron acceptor. The catalytic site of the hydrogenase is periplasmic and likely linked cytochrome *b* to the membrane. We provide evidence that the MetVF-type methylenetetrahydrofolate reductase is linked proteins MvhD and HdrCBA to the cytoplasmic membrane. Membrane preparations catalyzed the H_2_-dependent reduction of methylene-THF to methyl-THF. In our model, a transmembrane electrochemical H^+^ gradient is established by both scalar and vectorial protons that leads to the synthesis of 0.5 mol ATP/mol methylene-THF by a H^+^-F_1_F_o_ ATP synthase. This H_2_- and methylene-THF-dependent electron transport chain may be present in other cytochrome-containing acetogens as well and represents a third way of chemiosmotic energy conservation in acetogens, but only in addition to the well-established respiratory enzymes Rnf and Ech.

**IMPORTANCE** Acetogenic bacteria grow by making acetate from CO_2_ and are considered the first life forms on Earth since they couple CO_2_ reduction to the conservation of energy. How this is achieved has been an enigma ever since. Recently, two respiratory enzymes, a ferredoxin:NAD^+^ oxidoreductase (Rnf) and a ferredoxin:H^+^ oxidoreductase (Ech), have been found in cytochrome-free acetogenic model bacteria. However, some acetogens contain cytochromes in addition, and there has been a long-standing assumption of a cytochrome-containing electron transport chain in those acetogens. Here, we provide evidence for a respiratory chain in Sporomusa ovata that has a cytochrome-containing hydrogenase as the electron donor and a methylenetetrahydrofolate reductase as the terminal electron acceptor. This is the third way of chemiosmotic energy conservation found in acetogens.

## INTRODUCTION

Acetogenic bacteria are an ecophysiologically important group of strictly anaerobic bacteria; they are ubiquitous in nature and part of every anaerobic food web (1). Acetogens are of interest for the origin of life, since their metabolism is considered one of the oldest on Earth (2, 3). They have attracted much interest from the biotech industry recently, since they reduce the greenhouse gas carbon dioxide to biotechnologically interesting value-added compounds like acetate, ethanol (4–7), or even bioplastics (poly-β-hydroxybutyrate) (8, 9). Furthermore, acetogens can be used as catalysts in microbial hydrogen storage (10). Thus, they are prime candidates for a sustainable biotechnology. Acetogens grow at the thermodynamic limit of life and are model systems to explore the physiological basis for microbial growth at the thermodynamic equilibrium (11). Their characteristic feature is growth on and acetate production from H_2_ + CO_2_ (1). CO_2_ fixation is catalyzed by the Wood-Ljungdahl pathway (WLP), a two-branched linear pathway in which two molecules of CO_2_ are reduced and condensed with coenzyme A (CoA) to give acetyl-CoA, which is further converted to acetyl-phosphate and then acetate (12, 13).

The reduction of CO_2_ to acetyl-CoA requires eight electrons, carried by different electron carriers, which can be provided by molecular hydrogen or organic substrates. Thus, acetogens can grow chemoorganoheterotrophically with different carbon sources or chemolithoautotrophically with H_2_ + CO_2_. During lithotrophic growth, one ATP is synthesized in the acetate kinase reaction, but one ATP has to be invested for the activation of formate. Thus, net ATP synthesis by substrate-level phosphorylation is zero and an additional, chemiosmotic mechanism of ATP synthesis must exist. The ultimate part of a hypothetical respiratory chain, the ATP synthase, has been found in membranes of different acetogens (14). However, the nature of the respiratory enzyme(s) remained enigmatic for a long time (15). The first ion-translocating respiratory enzyme, the Rnf complex, a ferredoxin:NAD^+^ oxidoreductase, was discovered in 2010 in Acetobacterium woodii (16), and a second ferredoxin (Fd)-dependent respiratory enzyme, Ech (energy converting hydrogenase), a ferredoxin:H^+^ oxidoreductase, was discovered in another acetogen, Thermoanaerobacter kivui (17). Analysis of genome sequences available to date shows that Rnf and Ech are mutually exclusive in acetogens, and thus, any given acetogen has either Rnf or Ech but not both. Sporomusa ovata, the acetogen used in this study, has an Rnf complex.

It is important to note that these two types of respiratory enzymes are not directly involved in carbon flow in the WLP. This is somewhat surprising since the WLP has one reaction, the reduction of methylenetetrahydrofolate (methylene-THF) to methyl-THF, which has a fairly positive redox potential ([E_0_′] = −200 mV) (18), and its reduction with NADH (E_0_′ = −320 mV) or H_2_ (E_0_′ = −414 mV) is exergonic enough ([Δ*G*_0_′] = −23 or −41 kJ/mol) to drive ion transport out of the cell by a respiratory chain. Therefore, it was assumed already in 1977 (19) that methylene-THF may be the acceptor of a chemiosmotic electron transport chain, but evidence for this assumption could never be obtained. All the methylene-THF reductases (MTHFRs) that had been looked at were predominantly found in the cytoplasm and not at the membrane (20–25). However, it is important to note that the subunit composition of MTHFRs varies enormously in acetogens (24). Today, we can distinguish four different classes of MTHFRs in acetogens. The simplest type (the MetF-type) contains only one subunit, MetF, that binds and reduces methylene-THF using NADH as a cofactor (20). In the second type (the MetVF-type), MetF builds a heterodimer with an additional subunit, MetV. The MetVF-type is known to use electrons from reduced ferredoxin for methylene-THF reduction *in vitro* but has lost its ability to bind/oxidize NADH (24–26). MetV is a zinc binding protein with an FeS center that has a molecular mass of roughly 24 kDa. The third type of MTHFR builds a heterotrimer of MetVF and an RnfC-like subunit (RnfC2); RnfC2 is very similar to the NADH-oxidizing subunit of the Rnf complex, RnfC, and confers the use of NADH as an electron donor (23). In 2014, a fourth type (Hdr-type) of MTHFRs was proposed based mainly on genetic data. It is assumed that the MetVF core builds a hexaheteromeric complex with heterodisulfide reductase-like subunits HdrBCA and MvhD in Moorella thermoacetica, but the enzyme could not be purified and the mechanism of methylene-THF reduction remained unclear (22). Since HdrA(BC)-containing protein complexes are proposed to use the mechanism of flavin-based electron bifurcation (27–30), the MTHFR of M. thermoacetica was also proposed to transfer electrons from NADH to methylene-THF and a second, so-far-unknown electron acceptor. Similar to M. thermoacetica, S. ovata contains a gene cluster encoding an Hdr-type MTHFR (31).

We have followed up the question on the role of MTHFR in energy conservation and present evidence that the methylene-THF reductase of S. ovata is localized at the cytoplasmic membrane HdrCBA and MvhD. Membranes also contain a hydrogenase, and hydrogen oxidation is coupled to the reduction of methylene-THF and the generation of a H^+^ gradient across the membrane. These data are in accordance with a third way of chemiosmotic energy conservation in acetogens, in addition to the respiratory enzymes Rnf and Ech.

## RESULTS

### Bioinformatic analysis of methylene-THF reductase-encoding gene cluster.

**FIG 1:**
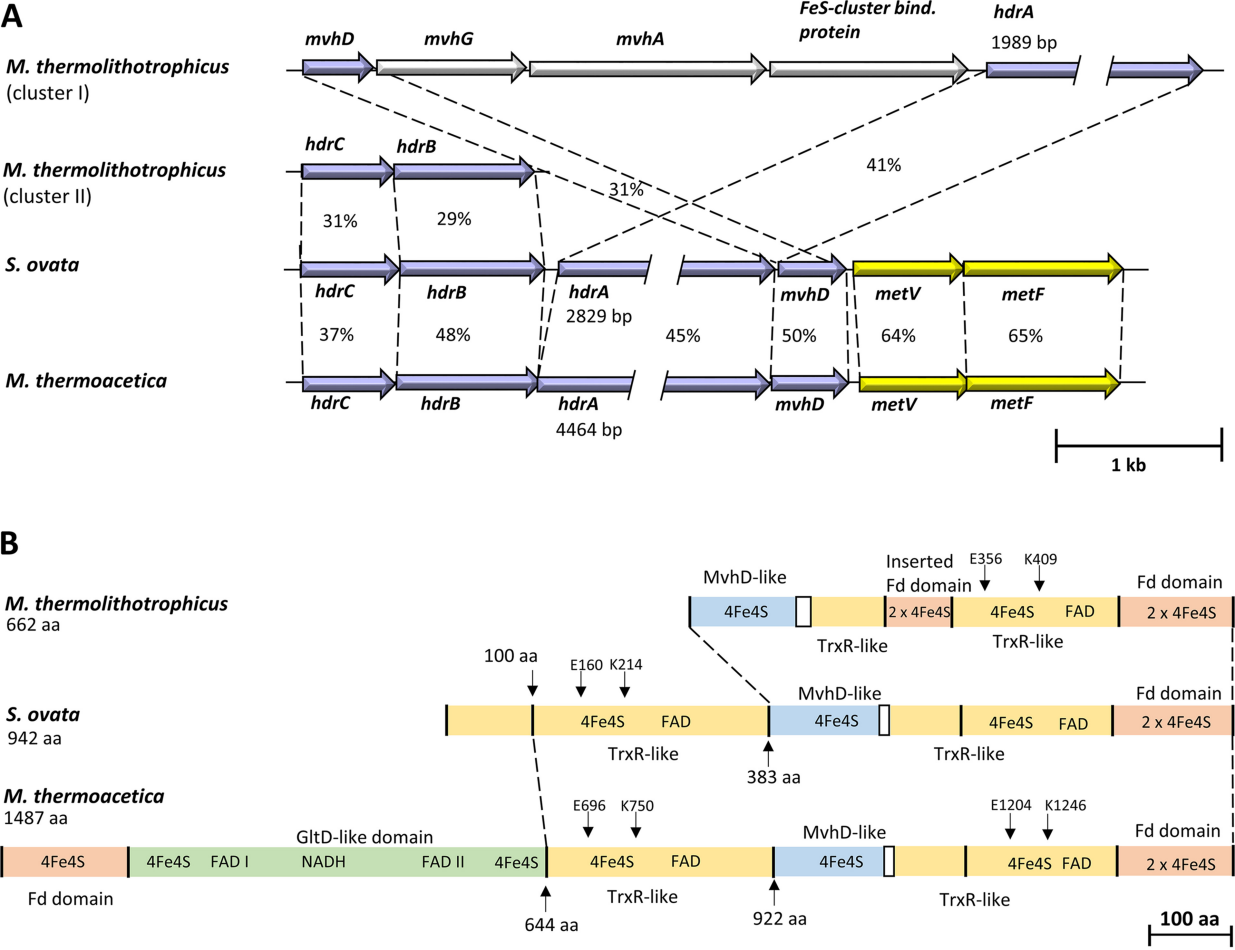
Bioinformatic analysis of the methylene-THF reductase of S. ovata. (A) Comparison of the methylene-THF reductase-encoding gene clusters of S. ovata and M. thermoacetica with the heterodisulfide reductase-encoding gene cluster of M. thermolithotrophicus. The Hdr of M. thermolithotrophicus is arranged in two distinct gene clusters. Identity between the single subunits on the protein level is given in percentages. *hdr*, heterodisulfide reductase; *mvh*, methyl viologen-reducing hydrogenase. (B) Comparison of HdrA of S. ovata, M. thermoacetica, and M. thermolithotrophicus. Conserved E and K residues, which indicate potential electron-bifurcating flavins, are displayed.

The core subunits MetV and MetF of the MTHFR from S. ovata are encoded by *SOV_1c07720* and *SOV_1c07730.* Upstream from these genes are genes that encode proteins similar to heterodisulfide reductase proteins HdrCBA and a methyl viologen (MV)-reducing hydrogenase (MvhD) (*SOV_1c07680–SOV_1c07710*) (24). The electron-bifurcating HdrCBA-MvhAGD complex of cytochrome-free methanogenic archaea like Methanothermococcus thermolithotrophicus (32) catalyzes electron bifurcation from hydrogen to the heterodisulfide of coenzyme M (CoM) and coenzyme B (CoB) (exergonic) and to ferredoxin (Fd) (endergonic). In M. thermoacetica, *hdrBCA-mvhD* form a transcriptional unit with *metVF*, and a hexaheteromeric MTHFR protein complex was proposed (22). The subunits HdrC, HdrB, HdrA, and MvhD of S. ovata are 31 and 37, 29 and 48, 41 and 45, or 31 and 50% identical to the corresponding subunits of M. thermolithotrophicus and M. thermoacetica, respectively (Fig. 1A). Furthermore, MetV and MetF of S. ovata are 64 and 65% identical to the homologous proteins of M. thermoacetica. Also, the predicted molecular masses of 22.2 (HdrC), 31.8 (HdrB), 14.4 (MvhD), 24.2 (MetV), and 35 (MetF) kDa are very similar to those of the homologous proteins of M. thermolithotrophicus and M. thermoacetica. Sequence alignments demonstrate highly conserved cofactor binding sites within the homologous proteins. Two [4Fe4S] clusters can be predicted in HdrC and HdrB, one [2Fe2S] cluster in MvhD, two [4Fe4S] clusters in MetV, and one flavin binding domain in MetF. Contrary to the subunits mentioned above, not only do the HdrA homologs differ strongly in their predicted molecular masses of 101 kDa in S. ovata, 162.8 kDa in M. thermoacetica, and 71 kDa in M. thermolithotrophicus but the alignments of the amino acid sequences of HdrA from S. ovata, M. thermoacetica, and M. thermolithotrophicus reveal two major differences. First, the C-terminal amino acid sequences of HdrA from S. ovata (amino acids [aa] 383 to 942) and M. thermoacetica (aa 922 to 1487) are very similar to the homolog of M. thermolithotrophicus and contain an MvhD-like domain, a thioredoxin reductase (TrxR)-like domain, and a C-terminal Fd domain, but the inserted Fd domain that is responsible for Fd binding in the bifurcating HdrABC-MvhAGD complex (32) is missing (Fig. 1B). Second, the N terminus of HdrA from M. thermoacetica contains an additional Fd domain and an additional TrxR-like domain, interrupted by an [4Fe4S] cluster-coordinating, FAD and NAD(P)H binding GltD domain. HdrA of S. ovata, however, only contains one additional TrxR-like domain (Fig. 1B). Altogether, eight [4Fe4S] clusters, three FAD binding sites, and one NAD(P)H binding site are predicted in HdrA of M. thermoacetica, whereas in HdrA of S. ovata, only five [4Fe4S] clusters and two FAD binding sites are predicted (Fig. 1B). HdrA is known as the flavin-containing subunit of potential electron-bifurcating enzymes, but nonbifurcating enzyme complexes with HdrA homologs also exist (27). The glutamate and lysine residues E356 and K409 of HdrA from M. thermolithotrophicus are conserved in bifurcating HdrA homologs and are used to differentiate between a bifurcating and a nonbifurcating flavin in HdrA (27). In HdrA of S. ovata, two flavin binding TrxR-like domains are present, but only in one are the E and K residues conserved (E160 and K216) (Fig. 1B), pointing toward a possible function of the complex in electron bifurcation. Contrarily, in M. thermoacetica, the E and K residues are conserved in both TrxR-like domains (E696 and K750 and E1204 and K1246) (Fig. 1B). Transmembrane helices could not be found in either of the subunits.

### Purification of the methylene-THF reductase.

**FIG 2:**
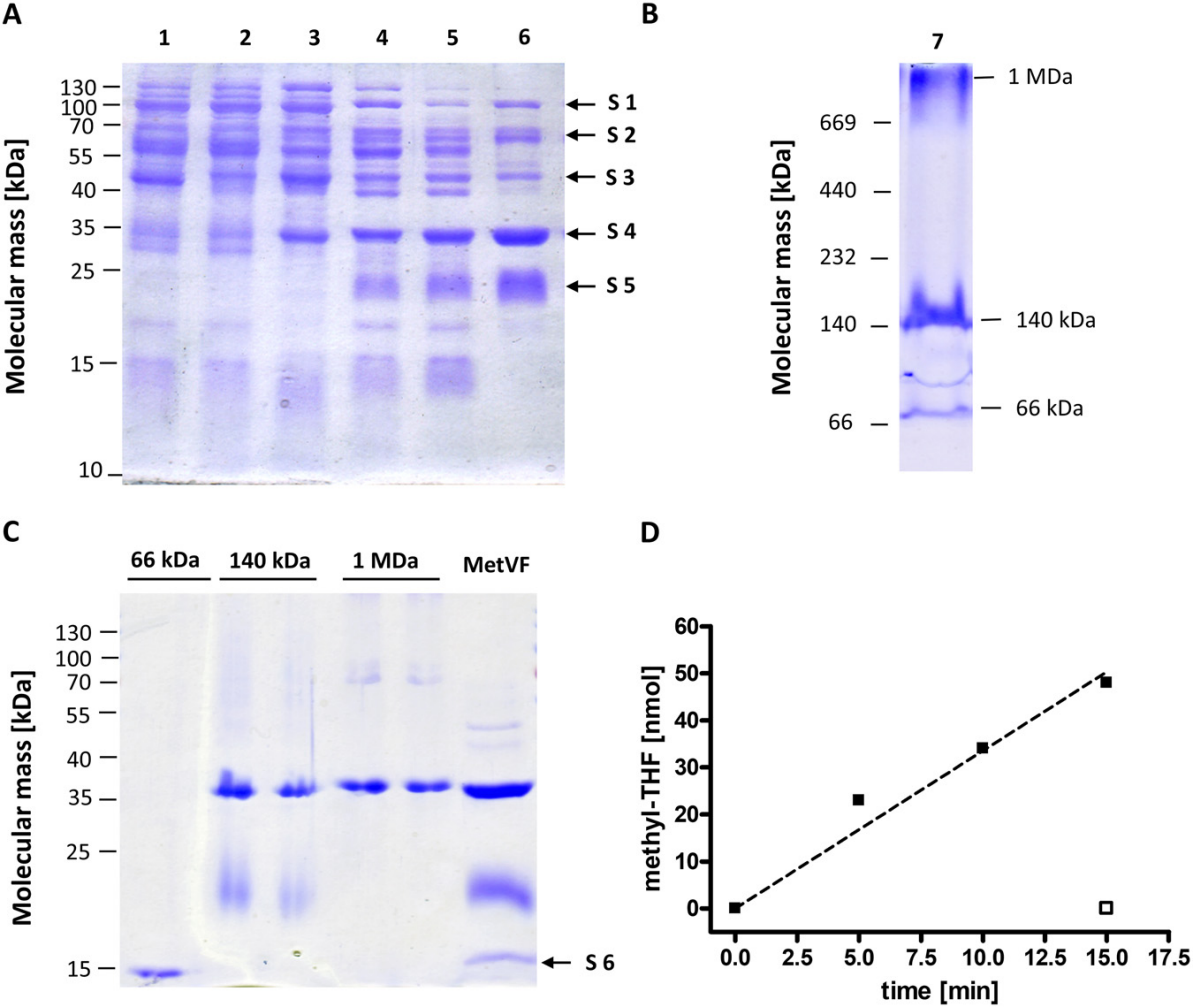
Purification and activity of the MetVF complex of Sporomusa ovata. (A) Amounts of 10 μg of protein sample from each purification step were separated in a denaturing SDS-PAGE. Lanes: 1, crude extract; 2, cytoplasmic fraction; 3, combined fractions after anionic exchange chromatography on Q Sepharose; 4, combined fractions after hydrophobic interaction chromatography on phenyl Sepharose; 5, combined fractions after anionic exchange chromatography on Q Sepharose HiTrap fast flow column; 6, combined fractions after gel filtration on Superdex 200 Increase column. Another batch of MetVF preparation (7) was separated by native PAGE under anoxic conditions (B), and the visible protein complexes with molecular masses of 1 MDa, 140 kDa, and 66 kDa were cut out of the gel and further separated in a denaturing SDS-PAGE (C) together with 10 μg of purified MetVF. MALDI-TOF MS analysis of protein complexes indicated as S1 to S6 (samples 1 to 6) is presented in Table S2. (D) Fd_red_-dependent methyl-THF formation from methylene-THF by purified MetVF. The assay contained 8.2 μg purified MetVF, and the amount of methyl-THF formed (black squares) was analyzed by HPLC as described previously (25). As a control, ferredoxin was omitted (open square).

To purify the methylene-THF reductase from S. ovata, the cytoplasmic fraction from fructose-grown cells was used. Since the crude extract did not catalyze methylene-THF-dependent oxidation of either NADH or NADPH, the MTHFR activity was traced by measuring the methylene-THF-dependent oxidation of reduced MV (MV_red_). The MV_red_:methylene-THF oxidoreductase activity was enriched 101-fold, from 5.1 U/mg to 513.9 U/mg, as shown by the results of fast liquid chromatography on Q Sepharose and phenyl Sepharose and gel filtration on a Superdex 200 Increase column (Table S1 in the supplemental material). Two major proteins with apparent molecular masses of 23 and 34 kDa, which fit the expected sizes of MetV (24.2 kDa) and MetF (35 kDa), became visible after separation of the enriched MV_red_:methylene-THF oxidoreductase on an SDS gel (Fig. 2A). The identities of MetV and MetF were confirmed by peptide mass fingerprinting (Table S2). Additionally, the preparation contained contaminating proteins (that were not found consistently) with molecular masses of ~100, 70, 45, 36, and 15 kDa. Matrix-assisted laser desorption ionization–time of flight mass spectrometry (MALDI-TOF MS) analysis identified these proteins as leucine-tRNA ligase, indolepyruvate oxidoreductase, AcsC and AcsD of the corrinoid/iron-sulfur protein (CoFeSP), and an ASC-1 homology (ASCH) domain-containing protein (Table S2). Separation of the MTHFR preparation in a native PAGE gel (Fig. 2B) revealed the presence of protein complexes with molecular masses of 1 MDa, 140 kDa, and 66 kDa. These complexes were cut out of the gel, denatured, and further separated by SDS-PAGE (Fig. 2C). The ~140-kDa complex was built by MetV and MetF but no other proteins. The 1-MDa complex was composed of a 36-kDa protein, which fits the mass of AcsD (Fig. 2C, Table S2), and the 66-kDa complex was shown to be composed of a protein with an apparent molecular mass of 15 kDa, which fits the mass of the ASCH-domain containing protein (Fig. 2C, Table S2). Apparently, HdrBCA-MvhD did not copurify with MetVF.

### Basic biochemical properties and activities catalyzed by MetVF.

Besides the methylene-THF-dependent oxidation of reduced MV, the purified MetVF complex catalyzed the methyl-THF-dependent reduction of MV, with an activity of 20 U/mg. Optimal conditions for the MV_red_:methylene-THF oxidoreductase activity were found at pH 6.5 and 40°C (Fig. S1A and S1B). MetVF did not catalyze methylene-THF-dependent NAD(P)H oxidation with or without the addition of oxidized ferredoxin. In contrast, methyl-THF was produced from methylene-THF (550 mU/mg) with reduced Fd (Fd_red_) (prereduced by the CO dehydrogenase [CODH] purified from A. woodii [33]) as the electron donor, whereas methyl-THF was not formed if Fd was omitted (Fig. 2D).

### Interaction of MetVF with the Hdr-like proteins.

From the experiments described above, it is evident that MetVF does not form a stable complex with Hdr- and Mvh-like proteins, as was previously reported for the MTHFR from M. thermoacetica (22). To detect possible weaker interactions, pulldown assays were performed. Therefore, a His or Strep tag-encoding sequence was added to the genes *hdrB*, *hdrA*, *mvhD*, *metV*, and *metF* and the genes were cloned into the vector pET21a for overproduction in E. coli BL21(DE3) Δ*iscR* (34, 35). The overproduced proteins were bound to the respective affinity matrix and incubated with crude extract of S. ovata. After removing nonspecifically bound proteins, the bait protein was eluted and the interaction partners were identified by Western blotting with antibodies generated against the overproduced and purified subunits. The immobilized proteins Strep-HdrB and His-HdrA interacted with MetF, as well as with the untagged HdrA and HdrB, respectively (Table 1, Fig. S2 and S3). The immobilized Strep-MvhD was shown to interact with the Hdr-like subunits HdrA and HdrB, as well as with MetF (Table 1, Fig. S4). Strep-tagged MetV interacted with HdrA and MetF, but HdrB could not be detected in Western blot analysis of the pulldown assay (Table 1, Fig. S5). The tagged variant MetF-His was clearly shown to interact with HdrA and HdrB (Table 1, Fig. S6). Even though the Hdr- and MvhD-like subunits could not be purified in a complex with MetVF and therefore do not form a tight complex with MetVF, it is apparent from these experiments that the subunits MetVF physically interact with the Hdr-like and MvhD-like proteins.

**TABLE 1:** Interactions of potential MTHFR subunits determined by pulldown assays

Bait protein	Occurrence of interaction with indicated prey protein^*a*^				
	HdrB	HdrA	MvhD	MetV	MetF
Strep-HdrB		Yes	ND	ND	Yes
His-HdrA	Yes		ND	ND	Yes
Strep-MvhD	Yes	Yes		ND	Yes
Strep-MetV	No	Yes	ND		Yes
MetF-His	Yes	Yes	ND	ND	

a Interactions between the proteins were analyzed (see Fig. S2 to S6). ND, not determined, since the interactions of MvhD or MetV with the other proteins were already identified using the tagged variants “Strep-MvhD” or “Strep-MetV”; yes, interaction detected; no, no interaction detected. Interactions with HdrC could not be determined, since an anti-HdrC antibody was not available.

### Localization of the HdrCBA-MvhD-MetVF complex.

**FIG 3:**
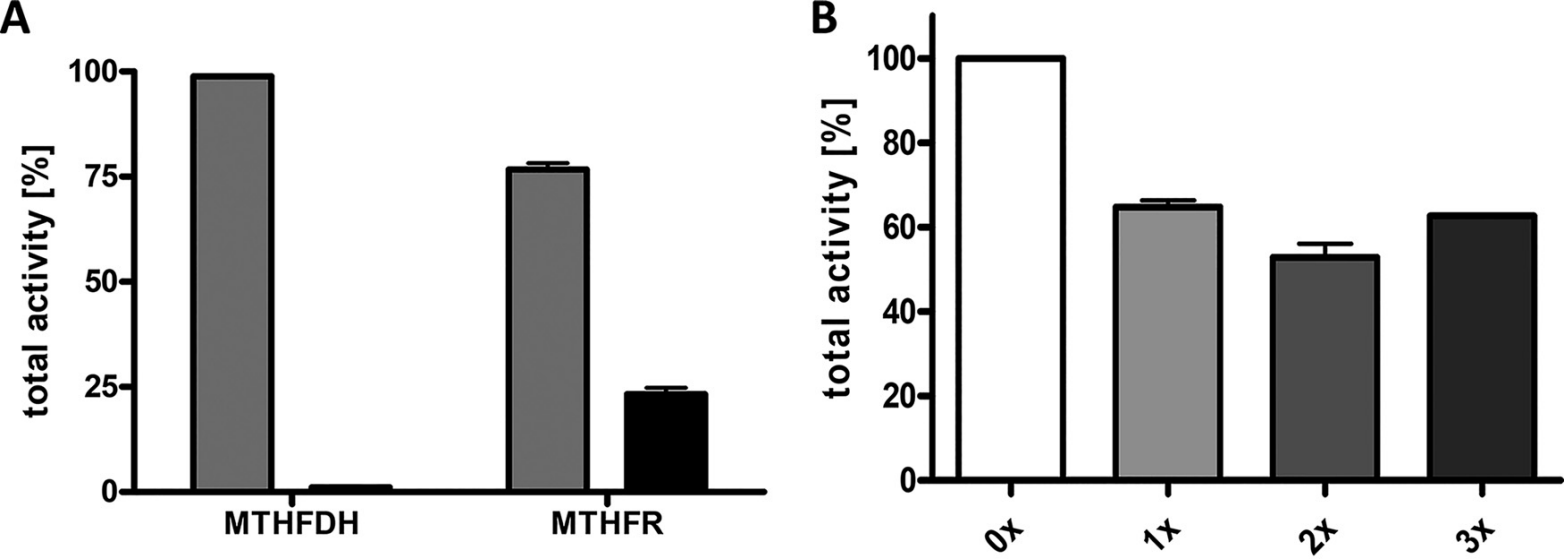
Cellular localization of the methylene-THF reductase and methylene-THF dehydrogenase activities. (A) The activities of MTHFR and MTHFDH were measured in the cytoplasm (gray bars) and (2 times) washed membrane (black bars). (B) The MTHFR activity was measured in unwashed (zero times [0×]) or washed (1×, 2×, or 3×) membranes. Activities of the cytoplasm plus membrane fractions (A) or unwashed membranes (B) were set to 100%. 100% corresponds to 687 U (A) and 283 U (B) for MTHFR and 3,459 U (A) for MTHFDH. Error bars show standard deviations.

If the MTHFR is the final enzyme of a chemiosmotic electron transport chain, it should be membrane bound or at least membrane associated. To address this question, cell extracts were separated into the soluble and particulate fractions and the MTHFR activities were determined. As a control, the methylene-THF dehydrogenase, which is a soluble enzyme (36–39), was also measured. Indeed, the MTHFDH activity was almost exclusively found in the soluble fraction (Fig. 3A). Whereas roughly 77% of the MTHFR activity was found in the cytoplasm, 23% of the activity was found in (2 times) washed membranes (Fig. 3A). Furthermore, the MTHFR activity could not be removed by washing the membranes (Fig. 3B), indicating that the MTHFR-membrane interaction is quite strong. These findings demonstrate that the major amount of MTHFR is located in the soluble fraction but that it is also associated with the membrane to a significant extent.

If HdrBCA-MvhD subunits anchor the MetVF complex to the membrane, they should also be localized, at least to some extent, at the cytoplasmic membrane. Since there is no activity assay for HdrBCA or MvhD, their localization was determined by Western blotting. Around 17 to 40% of HdrA, HdrB, MetV, and MetF proteins were found in (3 times) washed membranes; only MvhD was detected in the membrane fraction in a significantly larger amount of 75% (Table 2, Fig. S7A to E). Therefore, we assume that MvhD is the linker of the MTHFR to the membrane.

**TABLE 2:** Distribution of potential MTHFR subunits between cytoplasmic and membrane fractions

Protein	% of protein^*a*^ in:		
	Crude extract	Cytoplasm	Membranes
HdrB	100	79	17
HdrA	100	69	27
MvhD	100	23	75
MetV	100	70	23
MetF	100	60	40

a The Western blot signals (see Fig. S7) were quantified by measuring their densities using the software ImageJ.

### Washed membranes catalyze electron transfer from hydrogen to methylene-THF.

**FIG 4:**
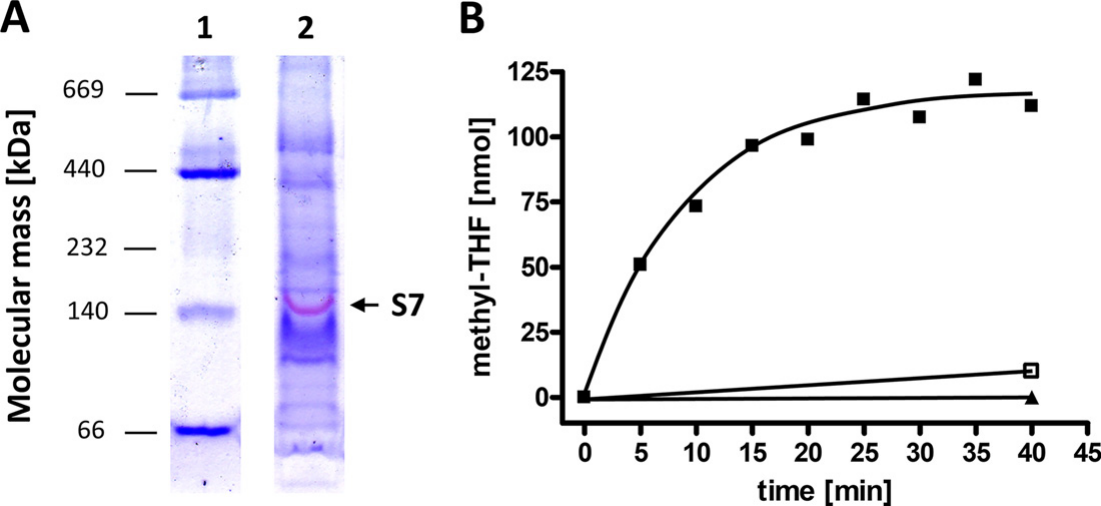
Hydrogen-dependent methylene-THF reduction in cytoplasmic membranes of S. ovata. (A) In-gel assay of hydrogenase activity of solubilized membrane protein. Amounts of 20 μg of solubilized membrane proteins were separated by native PAGE under anoxic conditions. Proteins with hydrogenase activity were stained (red, indicated as S7 [sample 7]) by incubation of the gel with triphenyltetrazolium chloride (0.4 μM) and methyl viologen (0.2 μM) under an atmosphere of 3% hydrogen in the anaerobic chamber before the gel was stained with Coomassie brilliant blue. The protein complex with hydrogenase activity (S7) was analyzed by MALDI-TOF MS (Table S2). (B) Formation of methyl-THF from methylene-THF by the cytoplasmic membrane of S. ovata with H_2_ as the electron donor. THF and formaldehyde were mixed in 1.8-mL anoxic glass cuvettes filled with reaction buffer to form methylene-THF. The gas phase was exchanged to 100% H_2_ (10^5^ Pa overpressure), and the reaction was started by the addition of 83 μg membrane protein. The reaction was stopped at the time points indicated, and the amount of methyl-THF formed was determined by HPLC (black squares). As controls, the assay was performed under 100% N_2_ (black triangle) or membrane protein was replaced with cytoplasmic protein (open square).

The localization of a part of the MTHFR at the cytoplasmic membrane is in line with the hypothesis that the MTHFR is the last link in a membrane-bound electron transport chain. But what could be the electron donor? Molecular hydrogen is a likely candidate, and therefore, we tested for membrane-bound hydrogenases by measuring the H_2_:MV oxidoreductase activity in washed membranes. Indeed, the membrane fraction catalyzed H_2_-dependent oxidized MV (MV_ox_) reduction with 24.9 U/mg. To identify the membrane-bound hydrogenase, we separated solubilized membrane proteins in a native PAGE gel and performed an in-gel hydrogenase activity assay (Fig. 4A). Only one protein complex with an apparent molecular mass of 140 kDa revealed H_2_:triphenyltetrazolium chloride (TTC) oxidoreductase activity and stained red in the gel, but nonspecific protein staining with Coomassie brilliant blue showed many more proteins present in the solubilizate. The complex was cut out of the gel and analyzed by MALDI-TOF MS for the proteins present (Table S2). Indeed, the large subunit HupL (SOV_1c08890) of a potential cytochrome *b-*containing periplasmic [NiFe] hydrogen uptake hydrogenase was present, as well as the large and small subunits, HydA2 and HydB2 (SOV_3c02090-SOV_3c02080), of a potential cytochrome *b*-containing periplasmic [FeFe] hydrogenase (Table S2).

S. ovata does not contain menaquinone but does contain ubiquinone (40), which could shuttle electrons from cytochrome *b* to the MTHFR. Indeed, washed membranes reduced the water-soluble quinone analogue anthraquinone-2,6-disulfonate (AQDS) with H_2_ as the electron donor with an activity of 1.8 U/mg (3.2 U/mg in cytoplasm). If the MTHFR is connected to the hydrogenase the quinone pool, AQDS should also be reduced by methyl-THF as the electron donor, and in fact, we found a methyl-THF:AQDS oxidoreductase activity of 271 mU/mg (100 mU/mg in cytoplasm). To indisputably demonstrate electron flow from the hydrogenase to the MTHFR in membranes of S. ovata, methylene-THF was added to the membrane fraction and incubated under a hydrogen atmosphere. Possible reduction of methylene-THF was monitored by using high-performance liquid chromatography (HPLC) to measure the methyl-THF produced. Methyl-THF was indeed produced by the membrane fraction in 10 independent experiments, with an average activity of 55 ± 37 mU/mg (mean ± standard deviation), whereas the cytoplasmic protein catalyzed methyl-THF production with only 5 ± 2 mU/mg (Fig. 4B). When the H_2_ atmosphere was replaced with CO or N_2_, no methyl-THF formation was detectable. Also, formate could not serve as the electron donor for methylene-THF reduction. These experiments clearly demonstrate a membrane-bound electron transport chain leading from molecular hydrogen to methylene-THF.

Since bioinformatic analysis indicated that one of the flavins of HdrA might be electron bifurcating, we tested for coreduction of NAD(P)^+^ or Fd_ox_ and methylene-THF, but no reduction of NAD(P)^+^ or Fd_ox_ was observed when methylene-THF was reduced with H_2_ as the electron donor and no significantly increased amounts of methyl-THF were formed. If the ubiquinone/ubiquinol couple (Q/QH_2_), which usually has a quite positive redox potential (E_0_′ [Q/QH_2_] = 69 to 110 mV) (41, 42), is the direct electron donor for methylene-THF reduction, the reaction becomes endergonic. Thus, electron confurcation rather than electron bifurcation is needed to run methylene-THF reduction. Therefore, we tested for cooxidation of NAD(P)H or Fd_red_ (reduced with the pyruvate:ferredoxin oxidoreductase [PFOR] of T. kivui [43]) and H_2_, but oxidation of either of the cofactors was not observed in the assays and no increased amounts of methyl-THF were produced in the assay. HdrB is known as the binding site of CoM-CoB in the heterodisulfide reductase (32), and therefore, we also tested disulfide-forming compounds as coreductants, but neither the addition of reduced glutathione (GSH_red_) nor the addition of dihydrolipoate resulted in an increased methyl-THF yield. Furthermore, we tested oxidized GSH (GSH_ox_) and lipoate as potential cooxidants for H_2_-dependent methylene-THF reduction, which also did not enhance the rate of methyl-THF formation.

### The bioenergetics of S. ovata relies on H^+^.

**FIG 5:**
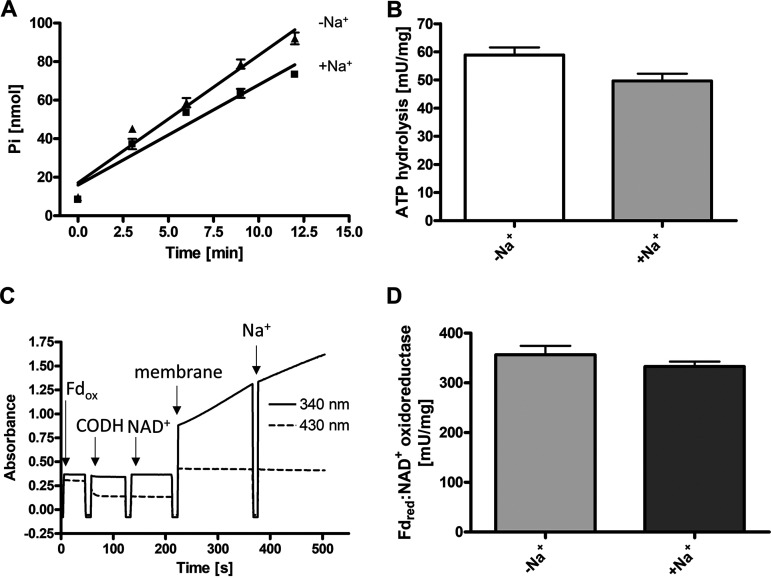
Na^+^ independence of ATPase and Rnf activities. (A) The ATPase activity of membranes from S. ovata was assayed in ATP hydrolysis buffer (100 mM Tris, 100 mM maleic acid, 5 mM MgCl_2_, pH 7.4), which was either supplemented with 20 mM Na^+^ (+Na^+^) or Na^+^ free (−Na^+^). The contaminating concentration of sodium in the Na^+^-free assay mixture was determined to be 198 μM. Membranes were added to the buffer and incubated at 30°C for 5 min. The assay was started by the addition of 3 mM Tris-ATP. ATP hydrolysis was detected by measuring free phosphate according to Heinonen and Lahti (52). (B) Mean ATP hydrolysis activity [mU/mg] was determined from four independent measurements of two biological replicates. (C) Fd_red_:NAD^+^ oxidoreductase activity of the membrane fraction was measured in Na^+^-free Rnf buffer (50 mM Tris, pH 7.7). A concentration of 30 μM Fd was added to the buffer and reduced with CO by the purified CO dehydrogenase (CODH) from A. woodii. The reduction of Fd was followed by measuring the absorbance at 430 nm. After the addition of 1 mM NAD^+^, the reaction was started by adding the membrane fraction to the assay. After 150 s, Na^+^ (20 mM) was added. (D) The mean activity of four independent measurements of two biological replicates of the experiment whose results are shown in panel C is given. To determine the mean activities of the Rnf complex in the presence of Na^+^, 20 mM Na^+^ was added before the assay was started. The concentration of Na^+^ in the Na^+^-free measurements was determined to be between 200 and 300 μM. Error bars show standard deviations.

The production of scalar protons in the periplasmic space might be coupled to ATP synthesis by an ATP synthase. In earlier studies, it was assumed that the respiratory chain of S. ovata was H^+^ dependent, but biochemical data were not presented (31). However, the *c* subunit of the F_1_F_o_ ATP synthase of S. ovata has the conserved Q…ET/S motif (44, 45) that is typical for Na^+^-dependent F_1_F_o_ ATP synthases. Therefore, we tested whether the ATP synthase requires Na^+^ for activity. Washed membranes hydrolyzed ATP with an activity of 59 mU/mg, and this activity was not stimulated by the addition of NaCl (Fig. 5A and B). The finding of Na^+^-independent ATPase activity was unexpected in light of the conserved Na^+^ binding site but expected if scalar protons were the driving force for ATP synthesis. Moreover, if the ATP synthase was H^+^ dependent, one would expect the Rnf complex of S. ovata to be H^+^ dependent as well. Indeed, Fd_red_-dependent NAD^+^ reduction was observed in the absence of NaCl and was not stimulated by the addition of NaCl to the buffer (Fig. 5C and D).

## DISCUSSION

In sum, our data point toward the existence of a membrane-integral electron transport chain that oxidizes molecular hydrogen (E_0_′ = −414 mV) in the periplasmatic space for the reduction of methylene-THF (E_0_′ = −200 mV) in the cytoplasmic space. A cytochrome-containing and membrane-associated hydrogenase is the first link in the electron transfer chain. Although only one protein complex showed hydrogenase activity in the native PAGE gel, MALDI-TOF MS analysis revealed the presence of subunits of two potentially periplasmic, membrane-associated, and cytochrome *b*-containing hydrogenases (HupSLC and HydA2B2C2). Indeed, S. ovata has two different *b*-type cytochromes with standard redox potentials of −153 ± 10 mV and −226 ± 14 mV (46). Closer inspection of the hydrogenase-encoding gene clusters revealed TAT sequences in the small subunits HupS (SOV_1c08880) and HydB2 (SOV_3c02080) (predicted with SignalP 5.0), indicating protein translocation into the periplasm. Subunits HupC and HydC2 resemble the diheme *b*-type cytochromes of the complexes. Cytochrome *b* is known as a membrane anchor of hydrogenases, as described for the [NiFe] hydrogenase of Wolinella succinogenes (HydABC) (47) and Sporomusa sphaeroides (HupSLC) (48). To figure out which of the hydrogenases (if not both) is responsible for hydrogen oxidation and electron transport to the MTHFR, a future task has to be the purification of the membrane-anchored hydrogenase(s). In any case, oxidation of hydrogen in the periplasmic space produces scalar protons, which are used as a driving force for ATP synthesis.

S. ovata is one of the few acetogens that has ubiquinone (and cytochromes) (40), and therefore, the further path of the electrons from cytochrome *b* the membrane to the electron acceptor, methylene-THF, may involve quinones as a second link. Consistent with their role as membrane-bound electron carriers in methylene-THF reduction, we observed reduction of the soluble quinone analog AQDS with H_2_ as the electron donor. Furthermore, AQDS was also reduced if methyl-THF was used as the electron donor, indicating a possible connection of the hydrogenase and the MTHFR AQDS. The involvement of a Q cycle in the electron transfer may result in an uptake of protons in the cytoplasm and a release of protons in the periplasmic space, further increasing the driving force of ATP synthesis. However, ubiquinones usually have redox potentials of 69 to 110 mV (41, 42), which are too electropositive to transfer electrons from ubiquinol (QH_2_) to methylene-THF (E_0_′ = −200 mV) (18). The energetic barrier may be overcome by electron confurcation with an additional reductant. HdrA of S. ovata has two flavin binding sites, and one of them has the typical signature for electron-bifurcating flavins (Fig. 1) (27, 32, 49). Furthermore, we clearly demonstrated physical interaction of MetVF with the HdrBCA and MvhD subunits by pulldown assays and the localization of the HdrBCA-MvhD-MetVF complex in the membrane fraction, but the membrane-integral interaction partner of the complex could not be identified. HdrA of M. thermoacetica is shown to oxidize NADH, and it is postulated that exergonic electron flow to methylene-THF is used to reduce a second, low-potential electron acceptor. HdrA of S. ovata also has NADH:oxidized benzyl viologen (BV_ox_) oxidoreductase activity, although with a lower activity of 0.5 U/mg compared to 190 U/mg in M. thermoacetica. In S. ovata, oxidation of NADH may be used to drive the endergonic reduction of methylene-THF with reduced quinones as the electron donor. Contrary to our hypothesis, NADH was not oxidized when added to the H_2_:methylene-THF oxidoreductase assay, and therefore, it might be possible that a coreductant other than NADH is needed. HdrBC is known as the binding site of the heterodisulfide in Hdr of methanogens (32); it is imaginable that (proteinogenic) dithiols might be the coreductant. However, neither dihydrolipoate nor GSH_red_ could enhance the rate of methyl-THF formation, and therefore, the participation of a potential dithiol-containing coreductant remains uncertain.

It is interesting to note that another potentially electron-bifurcating complex somewhat similar to the HdrBCA-MvhD-MetVF complex, the benzoyl-CoA reductase of Geobacter metallireducens, is also attached to the membrane (49). Its HdrA homolog also contains two conserved flavin binding sites, both of which have the electron bifurcation signature and, therefore, it is proposed that this complex catalyzes two tandem electron bifurcation reactions. Electrons, ultimately deriving from ferredoxin, are split once, to the exergonic branch (to reduce benzoyl-CoA) and to an endergonic branch, in which they are split a second time for the reduction of NAD^+^ (endergonic) and menaquinone (exergonic) (49).

**FIG 6:**
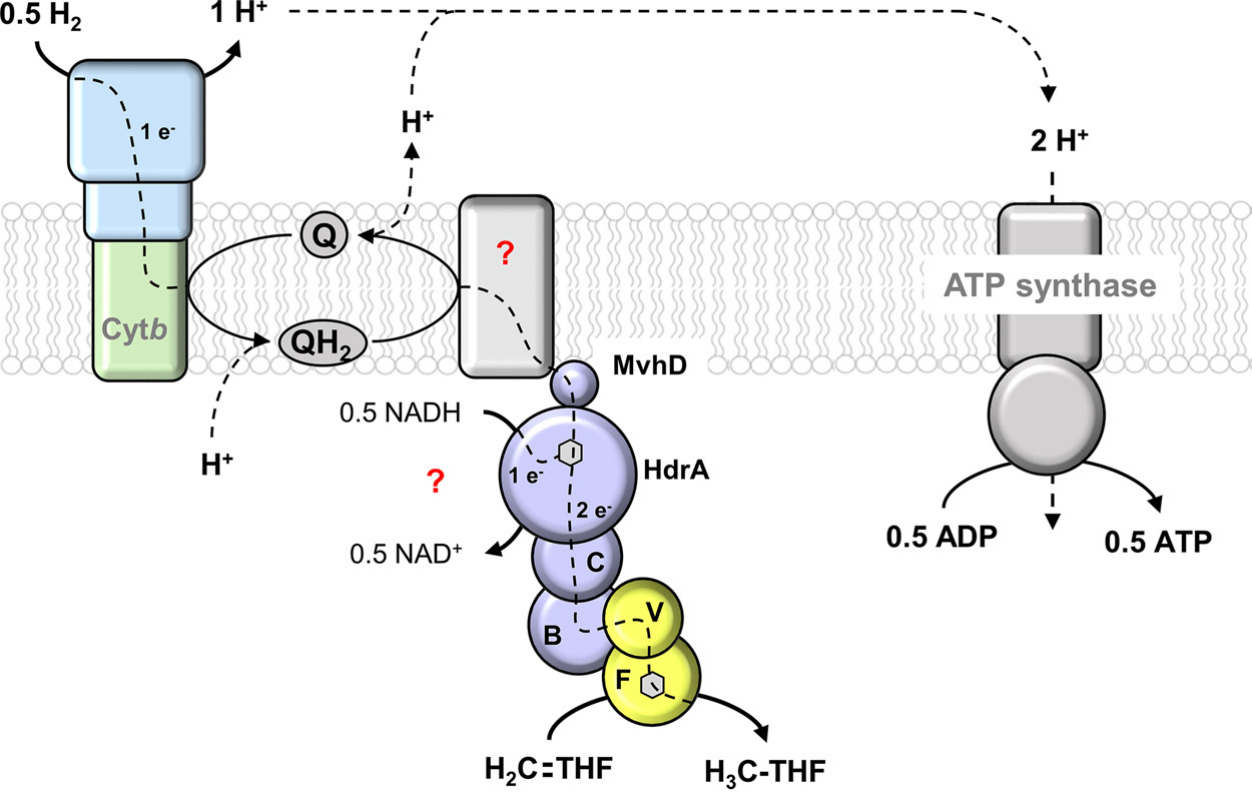
Model of hydrogen-dependent methylene-THF reduction associated with membrane-integral electron transport in S. ovata. The cytochrome *b*-containing hydrogenase oxidizes H_2_ in the periplasm, leading to the formation of scalar protons. Electrons are transferred ubiquinone and MvhD to the NADH-oxidizing subunit HdrA and from there to methylene-THF. It is also possible that electrons from H_2_ and NADH are confurcated by the bifurcating flavin of HdrA and further transferred HdrCB (C and B) to the core subunits MetV and MetF (V and F), where methylene-THF reduction occurs. The scalar protons of hydrogen oxidation and vectorial protons translocated by the quinone cycle subsequently drive ATP synthesis by the ATP synthase. The nature of the hypothetical Q cycle protein (question mark) remains to be identified.

The reduction of methylene-THF (E_0_′ = −200 mV) with H_2_ (E_0_′ = −414 mV) is exergonic (41.3 kJ/mol) and could be coupled to the translocation of 2.4 H^+^ (according to the equation Δ*G* = −*n* × F × μ̃_ion_, where *n* is the number of ions translocated, F is the Faraday constant [96.5 kJ mol^−1^ V^−1^], and μ̃_ion_ is the membrane potential [assumed to be −180 mV]). In our model, oxidation of 0.5 H_2_ in the periplasm produces one scalar proton (Fig. 6). Combined with the translocation of one proton by the Q cycle, a ΔH^+^ of 2 is generated. Hence, 0.5 mol ATP/mol methylene-THF reduced can be synthesized by the H^+^-dependent F_1_F_o_ ATP synthase (assuming a H^+^/ATP stoichiometry of 4) (Fig. 6). To further validate the model of hydrogen-driven ATP synthesis by methylene-THF reduction, the establishment of a protocol for the generation of inverted membrane vesicles is needed.

S. ovata belongs to the group of methylotrophic acetogens (50). During methylotrophic acetogenesis, the methyl group of the substrate is fed into the WLP at the level of methyl-THF. To generate electrons for CO_2_ reduction in the carbonyl branch of the WLP, methyl-THF has to be oxidized. Methyl group oxidation with NAD^+^ (E_0_′ = −320 mV) or Fd_ox_ (E_0_′ = ~−450 mV) is highly endergonic and therefore builds an energetic barrier (Δ*G*_0_′ = +23 or +48 kJ/mol). Our model also explains how this barrier is overcome. With ubiquinone and NAD^+^ as direct electron acceptors of methyl-THF, methyl group oxidation becomes exergonic (Δ*G*_0_′ = −29 kJ/mol, with E_0_′ [Q/QH_2_] = 69 mV).

### Conclusion.

In sum, we propose a third way of chemiosmotic energy conservation in acetogens, exemplified in the cytochrome- and quinone-containing S. ovata. The methylene-THF reductase is loosely attached to the membrane MvhD and HdrCBA and receives electrons from molecular hydrogen cytochrome *b* and maybe quinones. This electron transport chain results in the generation of 0.5 ATP per mole of methylene-THF reduced. The reaction is bidirectional and serves to drive endergonic methyl group oxidation during growth on methanol.

## MATERIALS AND METHODS

### Cultivation of S. ovata.

Sporomusa ovata was cultivated in a volume of 20 L as described previously (31), with 20 mM fructose as the carbon and energy source.

### Preparation of cytoplasm and membrane fractions.

Cytoplasm and membranes were prepared as described previously (31). If the cytoplasm was used for purification of the MTHFR, the cells were resuspended in buffer A (50 mM Tris/HCl, 20% glycerol, 20 mM MgSO_4_, 5 μM FAD, 2 mM dithioerythritol [DTE], 4 μM resazurin, pH 8.8) before cell disruption; otherwise, the pH of buffer A was 7.5.

### Purification of the methylene-THF reductase.

The cytoplasmic fraction was applied to a Q Sepharose HP column, which was equilibrated with buffer A (pH 8.8) and eluted with a linear gradient of 0 to 10% buffer B (buffer A plus 1 M NaCl, pH 8). The MTHFR eluted around 50 mM NaCl, and fractions with MV_red_:methylene-THF oxidoreductase activity were pooled. (NH_4_)_2_SO_4_ was added to a final concentration of 1.22 M, and the precipitate was removed by centrifugation (24,000 × *g* for 30 min). The supernatant was applied to a phenyl Sepharose HP column equilibrated with buffer C [buffer A plus 1.22 M (NH_4_)_2_SO_4_, pH 8]. The column was washed with 35% buffer D (buffer A at pH 8), and elution was performed with a linear gradient of 35 to 60% buffer D. The activity eluted around 650 mM (NH_4_)_2_SO_4_. Active fractions were pooled, desalted, and applied to a HiTrap Q fast-flow (FF) column, equilibrated with buffer D. Elution was performed with a linear gradient of 0 to 15% buffer B, and the MTHFR eluted at 75 mM NaCl. Active fractions were pooled and concentrated using ultrafiltration in 50-kDa Vivaspin tubes (Sartorius Stedim Biotech GmbH, Germany) applied to gel filtration on a Superdex 200 Increase column (10/300 GL; GE Healthcare). The column was equilibrated with buffer E (buffer A plus 250 mM NaCl, pH 7.5), and the MTHFR eluted at a volume of around 12.8 mL. The purified protein was stored at 4°C.

### Enzymatic activity assays.

All enzyme activities were measured in 1.8-mL anoxic glass cuvettes (0.5-cm diameter; Glasgerätebau Ochs, Germany) sealed by rubber stoppers. One unit is defined as the transfer of 2 μmol electrons min^−1^. Except where otherwise indicated, all measurements were performed at 30°C.

### (i) MV_red_:methylene-THF oxidoreductase, methyl-THF:MV oxidoreductase, and methylene-THF:NADP^+^ oxidoreductase.

The activities of the MTHFR and MTHFDH were measured as described previously (25, 31).

### (ii) H_2_- or Fd_red_-dependent methyl-THF formation.

The formation of methyl-THF from methylene-THF was performed routinely in buffer 1 (50 mM KPi (potassium phosphate), pH 7). The H_2_-dependent methyl-THF formation from methylene-THF by membranes of S. ovata was assayed at 30°C, whereas the reduced ferredoxin (Fd_red_)-dependent methyl-THF formation by the purified MTHFR was assayed at 40°C (the temperature optimum of the MTHFR). Methylene-THF was synthesized nonenzymatically as described previously (23). If Fd_red_ served as the electron donor, Fd_ox_ was prereduced by CO dehydrogenase (CODH; purified from A. woodii) (33) and methyl-THF formation was started by the addition of protein. If H_2_ served as the electron donor, the atmosphere was changed to 100% H_2_ (10^5^ pascals [Pa] overpressure) before the assay was started. After the reaction was stopped, the protein was separated from the sample by ultrafiltration (Vivaspin 500, 3-kDa cutoff; Sartorius Stedim Biotech GmbH, Goettingen, Germany) at 4°C and 12,000 × *g* for 40 min. Amounts of 10 μL of the protein-free samples were analyzed by HPLC on the Nucleodur reversed-phase (RP) C_18_ Gravity-SB column (3-μm particle size, 150 mm by 4.6 mm; Machery-Nagel) using isocratic elution with 33 mM KPi buffer (pH 3) containing 7% acetonitrile at a flow rate of 0.8 mL/min at 30°C. The absorbance at 313 nm was recorded, and a calibration curve of 0 to 10 nmol methyl-THF was used to calculate the amounts of methyl-THF produced.

### (iii) NAD^+^-dependent H_2_:Fd oxidoreductase.

The activity of the electron-bifurcating hydrogenase was measured as described previously (51), but only 30 μM Fd_ox_ was used and flavin was not added to the reaction buffer.

### (iv) NADH-dependent BV reduction.

Benzyl viologen (BV) reduction by HdrA with NADH as the electron donor was measured as described previously (22). Reduction of benzyl viologen was traced by measuring the increase in absorbance at 600 nm (ε = 10 mM^−1^ · cm^−1^).

### (v) H_2_:MV_ox_ oxidoreductase.

H_2_-dependent reduction of MV_ox_ was assayed in 50 mM KPi, 2 mM DTE, 4 μM resazurin, pH 7. The atmosphere was changed to 100% H_2_ (10^5^ Pa overpressure), 5 mM MV was added, and the measurement was started by the addition of the enzyme. The reduction of MV_ox_ was traced by measuring the absorbance at 604 nm (ε = 13.9 mM^−1^ · cm^−1^).

### (vi) Methyl-THF or H_2_:AQDS oxidoreductase.

Methyl-THF- or H_2_-dependent reduction of anthraquinone-2.6-disulfonate (AQDS) was measured in buffer 1 at 30°C. H_2_ (100%, 10^5^ Pa overpressure) or methyl-THF (0.2 mM) was used as the electron donor. The assay was started by the addition of AQDS (0.5 mM), and the reduction of AQDS was measured by following the absorbance at 408 nm (ε = 7.8 mM^−1^ · cm^−1^).

### (vii) Fd_red_:NAD^+^ oxidoreductase (Rnf activity).

To determine the ion dependence of the Rnf activity, the Fd_red_:NAD^+^ oxidoreductase activity was assayed in Na^+^-free buffer (20 mM Tris, pH 7.7). The contaminating concentration of Na^+^ in the Na^+^-free buffer was determined to be 104 μM. After the addition of the buffer to the cuvette, the gas atmosphere of the cuvette was changed to 100% CO (10^5^ Pa overpressure). An amount of 30 μM Fd_ox_ was added to the buffer and prereduced by the addition of 2 μg CODH. The membrane fraction was added, and the assay was started by the addition of NAD^+^ (1 mM). To determine the rate of NAD^+^ reduction, the absorbance was followed at 340 nm (ε = 6.3 mM^−1^ · cm^−1^). When the effect of Na^+^ was to be tested, 20 mM Na^+^ NaCl was added.

### (viii) ATP hydrolysis.

ATP hydrolysis was determined in Na^+^-free ATP hydrolysis buffer (100 mM Tris, 100 mM maleic acid, 5 mM MgCl_2_, pH 7.4). The contaminating concentration of sodium in the Na^+^-free assay buffer was 68.8 μM. If the effect of Na^+^ was to be tested, 20 mM NaCl was added. After the addition of the membrane fraction, the assay was started by the addition of 3 mM Tris-ATP. ATP hydrolysis was detected by measuring free phosphate according to Heinonen and Lahti (52).

### Heterologous production of the MTHFR subunits and generation of antibodies.

The DNA sequences encoding HdrB, HdrA, MvhD, MetV, and MetF were amplified by PCR (Table S3), and the resulting PCR products were cloned between the NdeI and BamHI restriction sites of the pET21a vector. The His or Strep tag-encoding sequences were added to either the 5′ or 3′ end of the open reading frames during amplification of the inserts (HdrB, HdrA, MvhD, and MetV), or the open reading frame was cloned in frame with the His tag-encoding sequence of pET21a (MetF). The constructs were subsequently used to transform E. coli HB101. Plasmids were isolated and verified by DNA sequencing and introduced into E. coli BL21(DE3) Δ*iscR*. Production and purification of the potential MTHFR subunits was performed as described previously (35). After affinity chromatography on Ni-nitrilotriacetic acid (NTA) or Strep-Tactin, the fractions containing HdrB, HdrA, MvhD, MetV, and MetF were pooled, concentrated to a volume of 300 μL, and applied to size exclusion chromatography on the Superdex 200 Increase column. The purified subunits were separated in SDS-PAGE, cut out of the gel, and used for immunization of rabbits (performed by Davids Biotechnologie, Regensburg, Germany) to generate antibodies against each subunit.

### Pulldown assays.

Bait proteins (tagged variants of MTHFR subunits) were overproduced in E. coli BL21(DE3) Δ*iscR* (see above), and the cells were harvested and passed through a French pressure cell at 110 MPa. After cell debris was removed, crude extracts were incubated for 30 min with the respective affinity ligand (Strep-Tactin or Ni-NTA). After unbound protein flowed through the column, the column was washed according to the manufacturer’s protocol (Machery Nagel, Düren, Germany, and IBA Lifesciences, Göttingen, Germany). Crude extract of S. ovata was added to the column-bound bait protein, and nonspecific proteins were removed by a second washing step according to the manufacturer’s protocol. Bait-prey protein complexes were eluted according to the manufacturer’s protocol.

### Immunological detection of MTHFR subunits.

The protein-containing samples were separated by denaturing SDS-PAGE according to Laemmli (53) and blotted to a nitrocellulose blotting membrane as described previously (54), followed by immunoblotting with a 1:2,000 dilution (anti-HdrA and anti-MetF antibodies) or a 1:500 dilution (anti-HdrB, anti-MvhD, and anti-MetV antibodies). Detection of primary antibodies was performed with a goat anti-rabbit IgG–horseradish peroxidase (HRP) conjugate (dilution of 1:10,000; Bio-Rad).

### Analytical methods.

The protein concentration was determined according to Bradford (55). Proteins were separated in 12% polyacrylamide gels according to Laemmli (53) or Schägger and von Jagow (56) and stained with Coomassie brilliant blue G250. Native PAGE was performed according to Wittig et al. (57).

### Biochemicals and enzymes.

NAD(P)^+^, NAD(P)H, FAD, FMN, methyl viologen, benzyl viologen, methyl-tetrahydrofolate, tetrahydrofolate, formaldehyde, and Tris-ATP were obtained from Sigma-Aldrich Chemie GmbH (Taufkirchen, Germany). Materials used for protein purification fast protein liquid chromatography (FPLC) were obtained from GE Healthcare, Sweden. Ferredoxin was purified from Clostridium pasteurianum (58), and CODH was purified from A. woodii (33).
