# Supplementary material for: A Third Way of Energy Conservation in Acetogenic Bacteria
Source: Microbiol Spectr. 2022 Jun 14;10(4):e01385-22. doi: 10.1128/spectrum.01385-22 (PMC9430772; doi:10.1128/spectrum.01385-22)
Supplement: Supplemental file 1 — Fig. S1 to S7 and Tables S1 to S3. Download spectrum.01385-22-s0001.pdf, PDF file, 0.6 MB [file spectrum.01385-22-s0001.pdf]

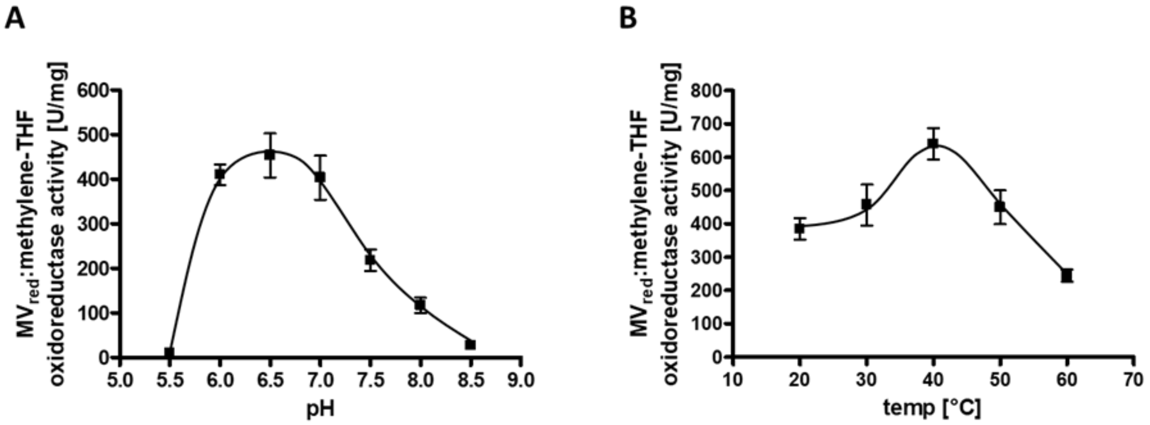

Fig. S1. pH- and temperature-dependence of MV$_{red}$:methylene-THF oxidoreductase activity of the purified MetVF complex. THF (500 µM) and formaldehyde (1.5 mM) were mixed in the assay buffer to generate methylene-THF, 5 mM MV$_{ox}$ were added and prereduced with sodium dithionite to an A_{604} of ~2. The assays were started by the addition of MTHFR. To analyze the pH dependence of the reaction, the assays were performed in 25 mM MES/MOPS/Tris/CHES buffer with different pH values, containing 10 mM NaCl, 20 mM $MgSO_4$, 2 mM DTE and 4 µM resazurin. (A). To determine the temperature dependence of the reaction, the assay was performed in MOPS buffer (50 mM MOPS, pH 7, 10 mM NaCl, 20 mM $MgSO_4$, 2 mM DTE, 4 µM resazurin). 100 % = 453 in (A) and 640.3 U/mg in (B).

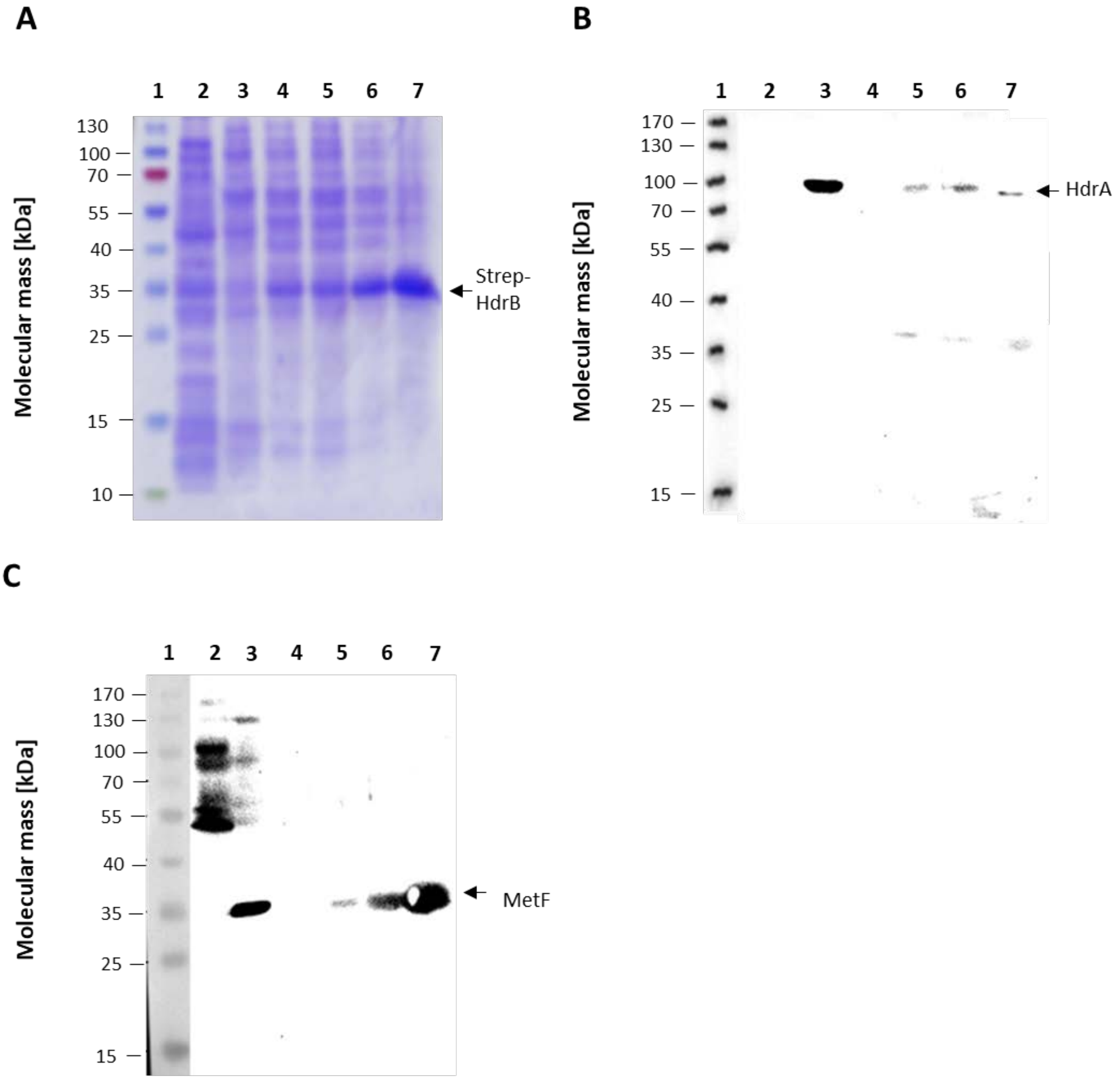

Fig. S2. Denaturating SDS-PAGE and Western blot of pull-down assays with Strep-HdrB as „bait“ protein. Strep-HdrB was overproduced in *E. coli* BL21 (DE3) Δ*iscR*, cells were harvested, disrupted and the resulting crude extract was added to a Strep-Tactin matrix. The column was washed according to the manufacturer's instructions. Crude extract of *S. ovata* was added and incubated with the column bound "bait" protein for 30 min. The column was washed again and HdrB-"prey" protein was eluted from the column according to the manufacturer's protocol. 50 µg of each protein fraction were separated in an SDS-PAGE (A), blotted to a nitrocellulose membrane and incubated with anti-HdrA (B) and anti-MetF antibodies (C). Detection of the primary antibody was performed with a goat anti-rabbit IgG-HRP conjugate. Lane 1, standard protein; lane 2, crude extract from *E. coli* BL21 (DE3) Δ*iscR*; lane 3, crude extract form *S. ovata*; lane 4, wash fraction after incubation of crude extract of *S. ovata* with „bait“ protein; lane 5-7 elution fractions 1-3.

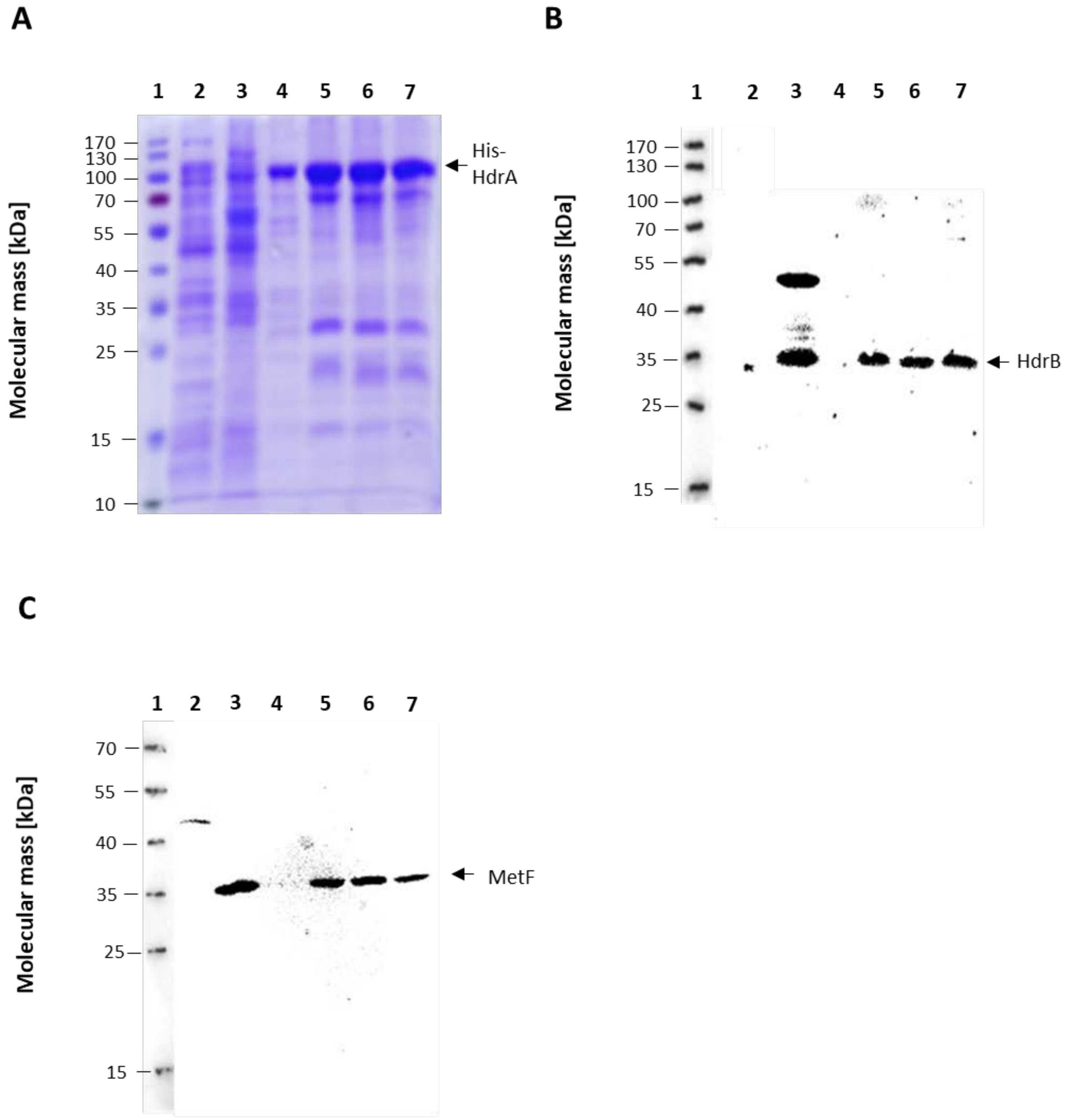

Fig. S3. Denaturating SDS-PAGE and Western blot of pull-down assays with His-HdrA as „bait“ protein. His-HdrA was overproduced in *E. coli* BL21 (DE3) ∆*iscR*, cells were harvested, disrupted and the resulting crude extract was added to a Nickel-NTA matrix. The column was washed according to the manufacturer’s instructions. Crude extract of *S. ovata* was added and incubated with the column bound “bait” protein for 30 min. The column was washed again and HdrA-“prey“ protein was eluted from the column according to the manufacturer’s protocol. 50 µg of each protein fraction were separated in an SDS-PAGE (A), blotted to a nitrocellulose membrane and incubated with anti-HdrB (B) and anti-MetF antibodies (C). Detection of the primary antibody was performed with a goat anti-rabbit IgG-HRP conjugate. Lane 1, standard protein; lane 2, crude extract from *E. coli* BL21 (DE3) ∆*iscR*; lane 3, crude extract form *S. ovata*; lane 4, wash fraction after incubation of crude extract of *S. ovata* with „bait“ protein; lane 5-7 elution fractions 1-3.

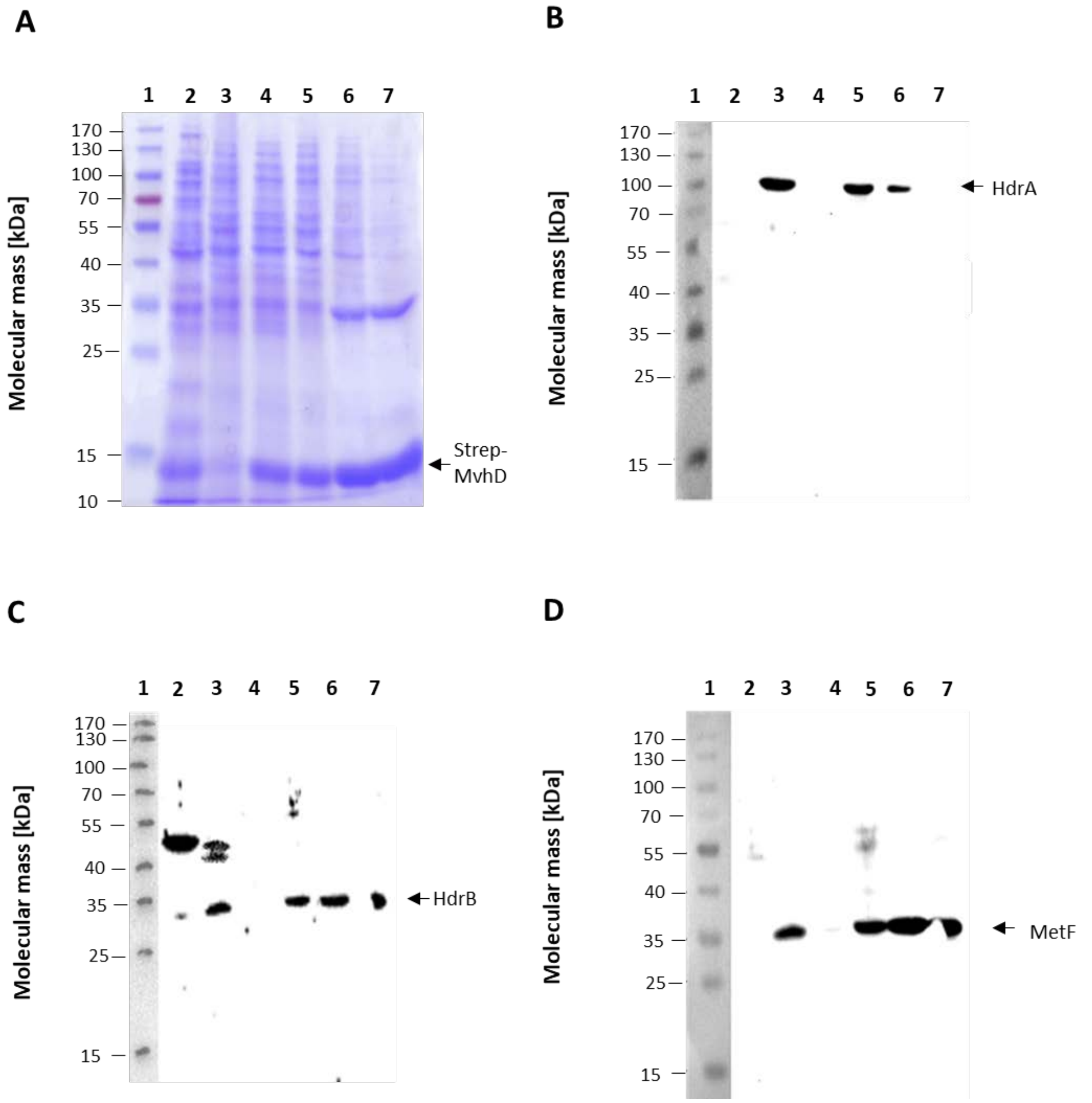

Fig. S4. Denaturating SDS-PAGE and Western blot of pull-down assays with Strep-MvhD as „bait“ protein. Strep-MvhD was overproduced in *E. coli* BL21 (DE3) Δ*iscR*, cells were harvested, disrupted and the resulting crude extract was added to a Strep-Tactin matrix. The column was washed according to the manufacturer’s instructions. Crude extract of *S. ovata* was added and incubated with the column bound “bait” protein for 30 min. The column was washed again and MvhD-“prey“ protein was eluted from the column according to the manufacturer’s protocol. 50 µg of each protein fraction were separated in an SDS-PAGE (A), blotted to a nitrocellulose membrane and incubated with anti-HdrA (B), anti-HdrB antibodies (C) and anti-MetF (D). Detection of the primary antibody was performed with a goat anti-rabbit IgG-HRP conjugate. Lane 1, standard protein; lane 2, crude extract from *E. coli* BL21 (DE3) Δ*iscR*; lane 3, crude extract form *S. ovata*; lane 4, wash fraction after incubation of crude extract of *S. ovata* with „bait“ protein; lane 5-7 elution fractions 1-3.

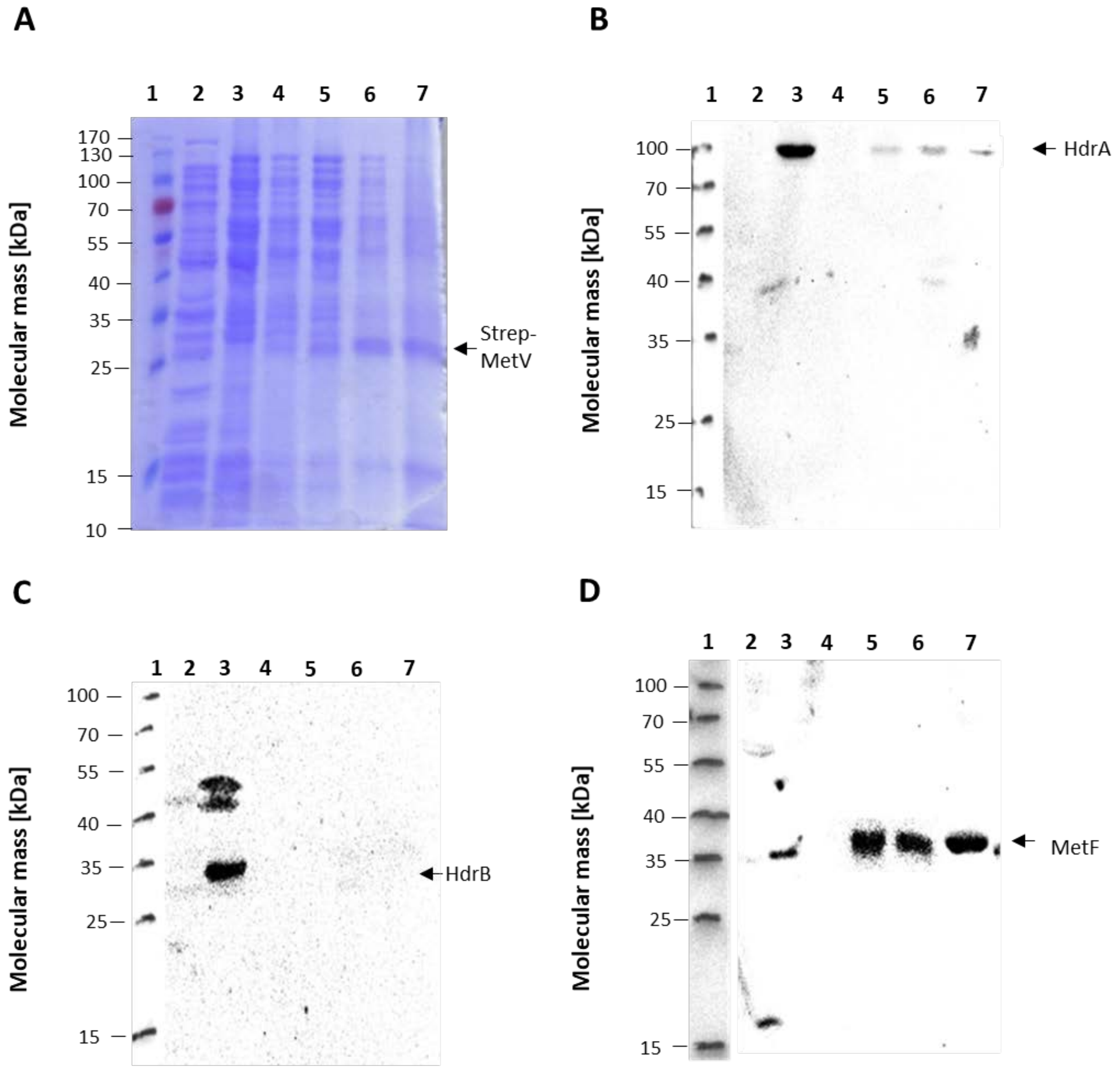

Fig. S5. Denaturating SDS-PAGE and Western blot of pull-down assays with Strep-MetV as „bait" protein. Strep-MetV was overproduced in *E. coli* BL21 (DE3) Δ*iscR*, cells were harvested, disrupted and the resulting crude extract was added to a Strep-Tactin matrix. The column was washed according to the manufacturer's instructions. Crude extract of *S. ovata* was added and incubated with the column bound "bait" protein for 30 min. The column was washed again and MetV-"prey" protein was eluted from the column according to the manufacturer's protocol. 50 µg of each protein fraction were separated in an SDS-PAGE (A), blotted to a nitrocellulose membrane and incubated with anti-HdrA (B), anti-HdrB antibodies (C) and anti-MetF (D). Detection of the primary antibody was performed with a goat anti-rabbit IgG-HRP conjugate. Lane 1, standard protein; lane 2, crude extract from *E. coli* BL21 (DE3) Δ*iscR*; lane 3, crude extract form *S. ovata*; lane 4, wash fraction after incubation of crude extract of *S. ovata* with „bait" protein; lane 5-7 elution fractions 1-3.

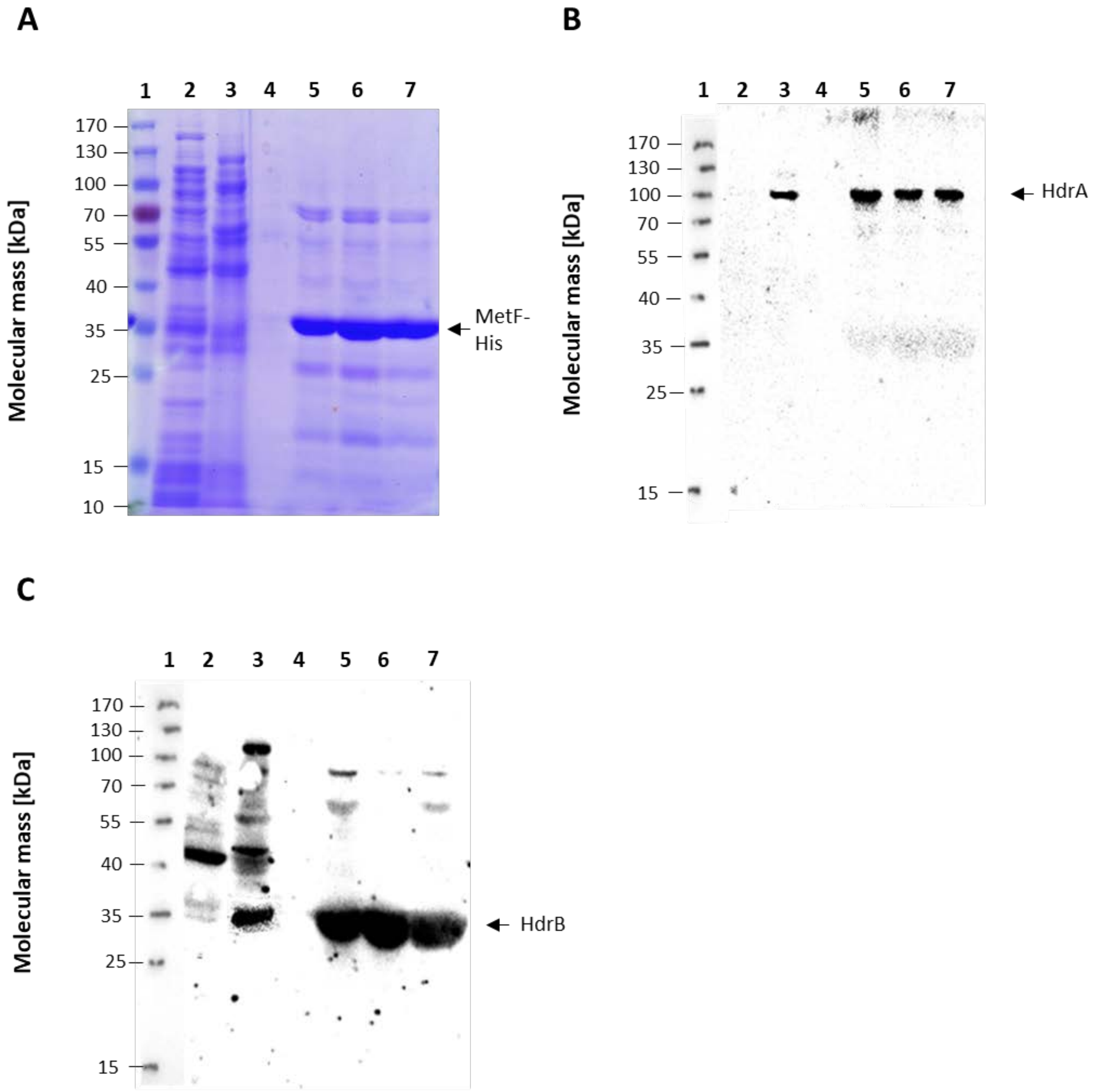

Fig. S6. Denaturating SDS-PAGE and Western blot of pull-down assays with MetF-His as „bait“ protein. MetF-His was overproduced in *E. coli* BL21 (DE3) Δ*iscR*, cells were harvested, disrupted and the resulting crude extract was added to a Nickel-NTA matrix. The column was washed according to the manufacturer’s instructions. Crude extract of *S. ovata* was added and incubated with the column bound “bait” protein for 30 min. The column was washed again and MetF-“prey“ protein was eluted from the column according to the manufacturer’s protocol. 50 µg of each protein fraction were separated in an SDS-PAGE (A), blotted to a nitrocellulose membrane and incubated with anti-HdrA (B) and anti-HdrB antibodies (C). Detection of the primary antibody was performed with a goat anti-rabbit IgG-HRP conjugate. Lane 1, standard protein; lane 2, crude extract from *E. coli* BL21 (DE3) Δ*iscR*; lane 3, crude extract form *S. ovata*; lane 4, wash fraction after incubation of crude extract of *S. ovata* with „bait“ protein; lane 5-7 elution fractions 1-3.

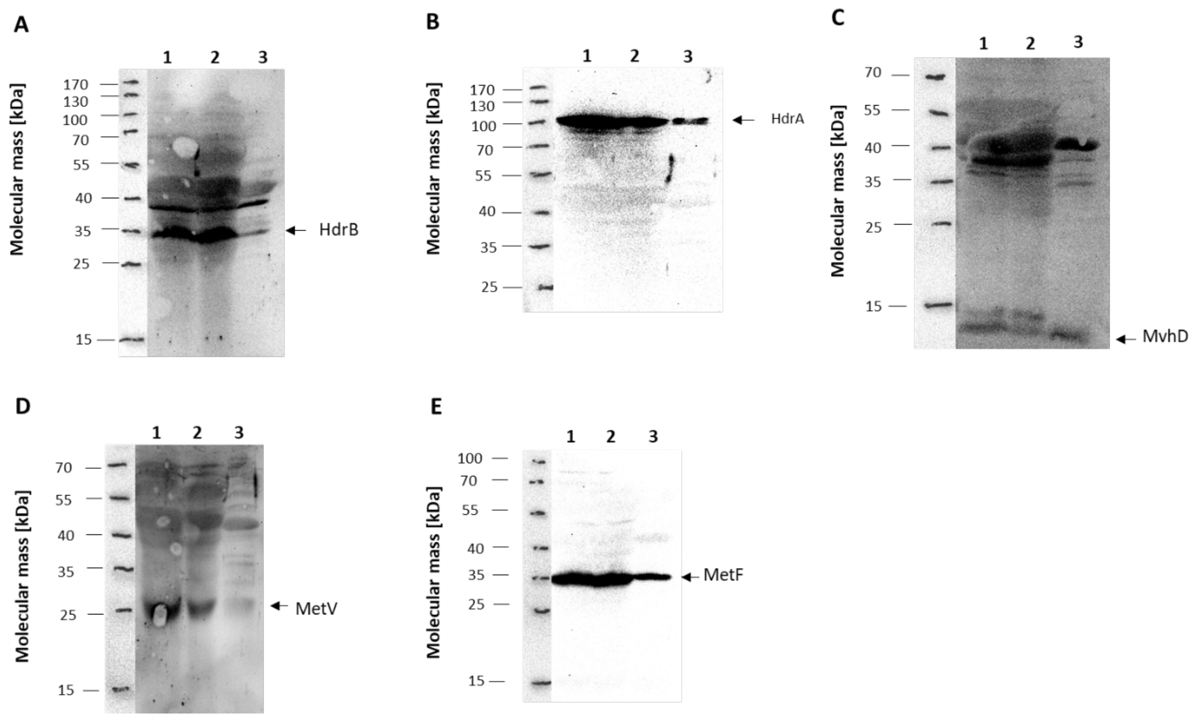

Fig. S7. Cellular localization of HdrBCA and MvhD. Equal volumes (10 µl) of crude extract (1), cytoplasm (2) and (3x) washed membranes (3) from *S. ovata* were separated in a denaturating SDS-PAGE and blotted onto a nitrocellulose membrane. The membranes were incubated with primary antibodies against HdrB (A), HdrA(B), MvhD (C), MetV (D) and MetF (E), washed and incubated with a secondary antibody (anti-rabbit IgG-HRP conjugate) to detect the potential MTHFR subunits.

Table S1. Purification of MetVF of *S. ovata.*

	Protein [mg]	MV_{red}:methylene-THF oxidoreductase [U*/mg]	Total activity [U]	Yield [%]	Purification [x-fold]
**Crude extract**	7101.00	5.1	36100	100.00	1.0
**Cytoplasm**	5835.06	6.3	36562	101.28	1.2
**Q Sepharose**	243.6	12.1	2950	8.17	2.4
**Phenyl Sepharose**	12.42	190.5	2366	6.55	37.5
**Q Sepharose**	4.18	243.3	1016	2.82	47.9
**Superdex 200**	0.56	513.9	288	0.80	101.2

*1 Unit is defined as 2 µmol methyl viologen oxidized per minute.

Table S2. Identification of proteins by MALDI-TOF MS. Proteins of the enriched MTHFR preparation (S1-S6 in Fig. 2A) and protein that showed hydrogenase activity in the in-gel assay (S7 in Fig. 4A) were cut out of the gel and analyzed by MADLI-TOF MS.

Sample	Accession no.	Annotation	Mass [kDa]	Protein identification probability [%]	Quantitative value (normalised total spectra)
**Sample 1 (100 kDa)**	**SOV_1c08840**	Leucine-tRNA ligase	94	100	1705
	**SOV_1c07850**	Valine-tRNA ligase	101	100	194
**Sample 2 (70 kDa)**	**SOV_2c04360**	Indolepyruvate OR, IorA	65	100	1112
	**SOV_5c03190**	electron transfer protein	65	100	292
	**SOV_1c01200**	Chaperone, DnaK	67	100	202
**Sample 3 (45 kDa)**	**SOV_6c02360**	Histidinol DH	48	100	635
	**SOV_1c07630**	AcsC	47	100	330
	**SOV_3c09870**	Methyltransferase	52	100	101
**Sample 4 (35 kDa)**	**SOV_1c07730**	**MetF**	35	100	229
	**SOV_1c07660**	AcsD	35	100	17
	**SOV_1c07720**	MetV	24	100	9
**Sample 5 (23 kDa)**	**SOV_1c07730**	MetF	35	100	25
	**SOV_1c07720**	**MetV**	24	100	214
**Sample 6 (15 kDa)**	**SPSIL_47490**	ASCH domain protein (*Sporomusa silvacetica*)	14	100	90
	**SOV_4c00100**	Cytidine deaminase	14	100	15
**Sample 7 (140 kDa)**	**SOV_1c08890**	[NiFe] hydrogenase large subunit, HupL	70	100	32
	**SOV_3c02090**	[FeFe] hydrogenase large subunit, HydA2	46	100	30
	**SOV_3c02080**	[FeFe] hydrogenase small subunit,HydB2	12	100	7

Table S3. Oligonucleotides used in this study. Tag encoding sequences are depicted in *italics*, restriction sites are underlined.

	**Oligonucleotides**		
**Construct**	**Forward (5'-3')**	**Reverse (5'-3')**	**Method**
**pET21a_Strep-HdrC**	TTTCATATG*tggagccacccgcagttcgaaaaatctgcg*ATGAAATTAGGCTATTATCCGGGATGTTCAC	TTTGGATCCTTACAACAGGTGCAATGATGTCAAGAGC	Classic ligation
**pET21a_HdrA**	TTTCATATGCAAAAGGATGTGCTCGTTATTG	TTTGGATCCTTAACCCACCTCCGTCAACG	Classic ligation
**pET21a_His-HdrA**	*caccaccac*ATGCAAAAGGATGTGCTC	*atgatgatg*CATATGTATATCTCCTTCTTAAAG	Blunt-end ligation (after PCR using pET21a_HdrA as template)
**pET21a_Strep-MvhD**	TTTCATATG*tggagccacccgcagttcgaaaaatctgcg*ATGAGTGATGTTAAAGTAGTAGGATTTGTTTGTCG	TTTGGATCCTAGGCCTTCCGGGCCTTAA	Classic ligation
**pET21a_MetF-His**	TTTCATATGAGCGAAGTAGCAATTGATAAAC	TTTGGATCCGATACTTTTGGCCTTGGTAAGAATCC	Classic ligation
**pET21a_Strep-MetV**	TTTCATATG*tggagccacccgcagttcgaaaaatctgcg*ATAGTTGCAGAAATCAAGCcgattg	TTTGGATCCTCATAACCGTACATCCTCCCTCAC	Classic ligation
